# Pretreatment Predictors of Response to PegIFN-RBV Therapy in Egyptian Patients with HCV Genotype 4

**DOI:** 10.1371/journal.pone.0153895

**Published:** 2016-04-21

**Authors:** Hanan H. Rizk, Nadia M. Hamdy, Nadia L. Al-Ansari, Hala O. El-Mesallamy

**Affiliations:** 1 Biochemistry Department, Faculty of Pharmacy, Ain-Shams University, Cairo, Egypt; 2 Endemic Medicine Department & Hepatology Unit, Faculty of Medicine, Ain-Shams University, Cairo, Egypt; Kaohsiung Medical University Hospital, Kaohsiung Medical University, TAIWAN

## Abstract

**Background:**

Egypt has the highest prevalence of a difficult to treat chronic hepatitis C virus (HCV), genotype 4. Pretreatment factors could guide individualization of therapy which aids in treatment optimization and *interleukin IL28B* gene polymorphism has been shown to closely relate to HCV treatment response. Polymorphisms in genes encoding inhibitors of T-cell response, which have role in disease progression as *Programmed Cell Death 1 (PD-1)*, and *Cytotoxic T-Lymphocytes Antigen-4 (CTLA-4)*, could be candidate markers predicting treatment response.

**Methods:**

This cohort study consisted of 200 chronic HCV genotype 4 infected patients treated with PegIFN α-2a and RBV in 2 hepatology centers. Genotyping of the polymorphisms in the *IL28B* gene region (rs12979860), *PD1*.*3* (rs11568821) and *CTLA-4* (rs231775) was performed on DNA collected from each patient using TaqMan^®^ genotyping assay. Groups were classified according to response into sustained virological responders (SVR), or non-responders (NR). A multivariate logistic regression analysis was used to identify potential markers, host pretreatment clinical and viral predictive factors including viral load, insulin resistance, and alpha fetoprotein (AFP) related to treatment response.

**Results:**

Our results showed that in a multivariate analyses *IL28B* C/C genotype was the most significant predictor for SVR (OR = 10.86; p<0.0001) followed by AFP (OR = 0.915; p = 0.001) then *CTLA-4*/G genotypes (OR = 1.948; p = 0.022). However, *PD-1*.*3*/A genotypes and platelets count were significantly related to response in univariate analysis only (OR = 1.973; p = 0.023; OR = 1.007; p = 0.009 respectively).

**Conclusion:**

*IL28B* SNP, AFP level, and *CTLA-4* SNP could be used in conjunction to predict treatment response in HCV genotype 4 infected Egyptian patients.

## Introduction

Hepatitis C virus (HCV) is a highly persistent human pathogen that infects the liver of 130–150 million patients worldwide, with yearly 3–4 million new infections, and 350,000 deaths due to all HCV-related causes [1, 2]. The largest viremic populations are in Egypt, with prevalence of almost 15% reaching staggering prevalence rates of approximately 20% reported in highly endemic areas, including urban centers and the Nile Delta [3, 4]. Moreover, 20% of worldwide HCV cases are of genotype 4, which constitutes 90% of HCV infections in Egypt [5].

Although successful use of direct-acting antivirals (DAAs) was recently reported in Western countries, the high cost, with HCV a socioeconomic condition hitting the poorest segments of the Egyptian population, limited trials and possible resistance which is still unknown on long terms represent a challenge to worldwide implementation [6]. Moreover, in Egypt new approved regimens of DAAs are combined with PegIFNα/RBV to minimize the development of viral breakthroughs and relapse due to resistance mutations [7]. Thus so far, the combined treatment with PegIFN-α+RBV remains a cornerstone and backbone in treatment for patients with chronic HCV in Egypt [8]. Nevertheless, fewer than half of patients are able to achieve SVR [9], defined as an undetectable HCV RNA level 6 months after treatment discontinuation [10]. Therefore, the prediction of SVR to IFN based therapy is still highly important to identify (i) patients with high chance to cure and therefore candidates for this therapy and (ii) those with low chance to respond to PEG-IFN+RBV, candidates for IFN free therapies. Moreover, deeper analysis of non-response may help elucidate its molecular mechanisms [11]. Treatment failure is likely to occur due to inherent viral and host factors such as the presence of certain SNPs and inappropriate drug regimens [12]. Genome-wide association studies (GWAS) identified SNPs in proximity to the promoter for the *IL28B* gene on chromosome 19, a human gene of host system innate antiviral defense which encodes IFN-lambda (λ), to be the most important pretreatment predictor of achieving SVR [13, 14]. Although *IL28* SNPs can be used in routine clinical practice for informing treatment decisions, yet *IL28B* genotype alone is not a perfect predictor of treatment outcome [15]. Moreover, studies that evaluated the role of this polymorphism in conjunction with other markers on genotype 4 especially in Egypt are still few [16].

Emerging studies show that T cell exhaustion occurring in viral infections correlates well with increased expression levels of inhibitory receptors including, PD-1 and CTLA-4 [17, 18]. Some genetic SNPs were found to cause changes in the expression of these molecules and thus modulating immune response [19, 20]. Previous studies have linked SNPs in *CTLA-4* gene to HCV severity and treatment outcome in HCV-1 genotype [21, 22]. Additionally, *PD-1*.*3/*A allele was denoted to be associated with SVR and to increase the predictive value of *IL28B* C/C genotype in HCV-1 and 3 genotypes [23].

Based on the aforementioned considerations, this study was conducted to investigate the association of; *IL28B* (rs12979860), *PD-1*.*3* (rs11568821) *and CTLA-4* 49A/G (rs231775) SNPs, pretreatment clinical factors as age, gender, insulin resistance, AFP, liver function tests and viral load, with the efficacy of PegIFNα+RBV treatment in Egyptian patients with chronic HCV genotype 4 infection.

## Patients and Methods

### Patients and Therapeutic protocol

A cohort of 200 consecutive Egyptian patients out of 230 collected cases with chronic HCV, positive for viral RNA, treatment naïve, and who were scheduled to receive combined double therapy with PegIFN/RBV since May 2013 were recruited from 2 hepatology centers; Yassin Abdulghafar center and Dr. Nadia Al-Ansary clinic, Cairo, Egypt. Thirty patients discontinued treatment either due to drug intolerance, adverse effects or non-compliance with 80/80/80 adherence rule and were thus excluded from the study. For inclusion, patients had to have HCV genotype 4, be aged >18 years, do not have history of alcohol abuse, lack co-infections, diabetes, thyroid dysfunction, autoimmune and other diseases. Diagnosis of chronic HCV infection was made by the persistence of anti-HCV antibodies and HCV-RNA over at least 6 months. Clinical and laboratory data of HCV patients at the time of diagnosis and prior to therapy were retrieved from their files, including HCV genotype and liver biopsy findings. Histopathological staging was done when feasible on 115 patients using METAVIR scoring system where (F) score representing fibrosis was staged from (F1-F3), and (F4) represents established cirrhosis, and (A) score represented activity (necroinflammation) with A1 mild, A2 moderate and A3 severe.

This study was approved by the local research ethics committee of faculty of Pharmacy Ain-Shams University, Yassin Abdulghafar center and Dr. Nadia Al-Ansary clinic, and a written informed consent was obtained from all participants in this study. The study was conducted in accordance with the provisions of the declaration of Helsinki and Good Clinical Practice guidelines.

#### Therapeutic protocol

Patients in this study were assigned to receive anti-HCV therapy with PEG-IFN α-2a plus RBV according to current guidelines: 180 μg (pegIFNα-2a)/week plus 800–1400 mg RBV given orally daily based on body weight for 24 or 48 weeks. Adequate follow-up and detection of serum HCV RNA was performed at the end of treatment and 6 months after the end of treatment. Duration of treatment was determined according to response recommendations and self reported adherence to treatment was ensured at weeks 4, 12, 24, and 48 and defined as taking at least 80% of each drug for at least 80% of the duration of therapy for all patients. Treatment was stopped only if a patient failed to achieve ≥ 2log reduction in viral load after 12 weeks.

Patients were classified according to response to treatment into 2 groups:

- Sustained virological responders (SVR): patients who were HCV RNA negative more than 6 months after the end of therapy.
- Non-responders (NR): included those who did not respond to treatment, stopped according to standard early virological stopping rules, had viral breakthrough (HCV-RNA reoccurrence during treatment after initial clearance) or had a relapse after end of treatment.

### Methods

#### Blood sample collection, storage and DNA extraction

Fasting blood (8 mL) was obtained from patients and samples were divided into aliquots of fresh serum and plasma samples for blood and chemistry testing, and aliquot of whole blood was collected into EDTA tubes for DNA extraction and subsequent genotyping assays.

#### Laboratory assessment

Complete blood count, liver function tests, serum AFP, fasting blood glucose and virological testing were performed at baseline and at each visit. Viral RNA was extracted from plasma using the QIAamp Viral RNA Mini Kit (Qiagen Hilden, Germany) according to the manufacturer’s protocol. HCV genotype was defined by the reverse line probe assay (Innolipa v.1.0, innogenetics, Ghent, Belgium) according to the manufacturer’s instructions. Serum HCV RNA quantification during treatment and follow up periods was done by using the reverse PCR (COBAS Amplicator; Roche) according to the manufacturer’s protocol. Determination of serum insulin (Monobind Inc.) using enzyme linked immunosorbant assay (ELISA) kits and following ELISA procedures were carried out according to the manufacturers' instructions. Insulin resistance was determined by the homeostasis model of assessment (HOMA) using the formula: fasting insulin (μIU/mL) x fasting blood glucose (mg/dL)/405 [24].

#### Genotyping of *IL28B* (rs12979680), *PD-1*.*3* (rs11568821) and *CTLA-4 49A/G* (rs231775) genes

Human genomic DNA was extracted from peripheral blood using the QIAamp DNA blood mini kit (Qiagen Inc) and stored at –20°C until genotyping was performed. Genotyping of the *IL28B* (rs12979860), *PD-1*.*3* (rs11568821) and *CTLA-4* 49 A/G (rs231775) genes was performed by real-time PCR using the TaqMan SNP genotyping assay and the StepOnePlus automatic instrument (Applied Biosystems), according to the manufacturer’s instructions.

#### Statistical analysis

IBM SPSS statistics (V. 22.0, IBM Corp., USA, 2013) was used for data analysis. Data were expressed as mean ± S.D for quantitative parametric measures, in addition to Median Percentiles for quantitative non-parametric measures and both number and percentage for categorized data. Student's *t* test was used for comparison of two independent groups for parametric data and Wilcoxon Rank Sum for non-parametric data. However, for comparison between more than 2 patient groups for parametric data we used analysis of variance (ANOVA) and Kruskall Wallis for non-parametric data. Treatment response rates in patients with different SNPs were analyzed using contingency tables. For statistical comparisons between the groups, Chi-square test, Fisher’s exact test and Mann–Whitney U test were used as appropriate. Hardy–Weinberg equilibrium was assessed in the study population. Covariates with a p < 0.05 at univariate analysis were included in a binary multivariate logistic regression model using step-forward selection method to evaluate possible independent predictors of treatment responses, and results were reported as odds ratio (OR) and their 95% confidence intervals (CIs). The area under the ROC curve indicated the prediction capacity of this analysis. For all tests, *p<* 0.05 was considered statistically significant.

## Results

### Characteristics of the study population

A total of 200 HCV-4 infected Egyptian patients, 71 (35.5%) women, and 129 (64.5%) men, were studied. According to the patients response to PEG-IFN and ribavirin therapy, 45.5% (n = 91) were classified as responders and 54.5% (n = 109) were classified as non-responders. The demographic and virological characteristics of the patients in the two groups are summarized in Table 1.

**Table 1:** Baseline demographic, clinical and virological characteristics of HCV infected study population.

Characteristics	SVR (n = 91)	NR (n = 109)	*p* value
**Age (years)**	50 ± 7.5	51.5 ± 8.5	0.244
**Male, n (%)**	61(67.0%)	68 (62.4%)	0.494
**Female, n(%)**	30 (33.0%)	41(37.6%)	
**BMI (kg/m2)**	23.8 ± 3.1	23.8 ± 2.9	0.879
**ALT (IU/L)** ^**Ω**^ **(N.V upto 45)**	120 (75–150)	100 (75–160)	0.806
**AST (IU/L)** ^**Ω**^ **(N.V upto 45)**	100 (70–130)	87 (70–150)	0.974
**Total bilirubin (N.V upto 1.2 mg/dl)**	0.95 ± 1.47	1.05 ± 0.55	0.189
**Leucocytes, X10**^**3**^ **(N.V = 4-7X10**^**3**^**)**	6.4 ± 1.9	6.2 ± 1.5	0.411
**Hemoglobin (N.V = M:13.5–17.5g%, F:12–15.5 g%)**	13.12 ± 1.4	13.45±1.4	0.1
**Platelets count, (10**^**9**^**/L) (N.V = 150–400)**	190.8 ± 55.7	170.0 ± 53.7	0.008*
**AFP (ng/ml)**^**Ω**^	3.3 (2.2–7)	7 (4–12)	<0.001*
**Total cholesterol (mg%) (N.V<200mg%)**	170 ± 30.2	181.2 ±25.3	0.2
**Fasting plasma glucose (mg/dl) (70–110 mg/dl)**	100 ± 16.9	102 ± 16	0.385
**Fasting serum insulin (μIU/ml) (N.V = 0.7–9 μIU/ml)**	11.4 ± 5.1	10.6 ± 4.9	0.22
**HOMA-IR (N.V<2)**	2.9 ± 1.6	2.7 ± 1.5	0.366
**Viral load (IU/ml)**			
**>400,000 n (%)**	41 (45.1%)	38 (34.9%)	0.142
**≥400,000 n (%)**	50 (54.9%)	71 (65.1%)	
**Liver histology (n = 115)**	n = 50	n = 65	
**Stage, F1/F2/F3/F4, n**	19/16/12/3	14/29/16/6	0.241
**Grade(n = 115), A1/A2/A3, n**	26/22/2	27/27/11	0.087
**Cirrhosis, n(%)**			
**Yes (20, 10%)**	5 (5.5%)	15 (13.8%)	0.06
**No (180, 90%)**	86 (94.5%)	94 (86.2%)	
**Treatment duration**			
**24 weeks (47)**	19	28	0.424
**48 weeks (153)**	72	81	
**RVR**^**¥**^			
**Yes (54)**	40 (44%)	14 (12.8%)	<0.0001
**No (146)**	51 (56%)	95 (87.2%)	

Data are expressed as mean ±SD

^Ω^ Data expressed as median (25^th^ and 75^th^ centiles-quartiles).

^¥^RVR: rapid virological response

*Denotes p-values below the level of 0.05 which are considered significant.

There were no significant differences in age, gender, and body mass index (BMI) among the two groups. Pretreatment platelets count was significantly higher among those who achieved SVR (P = 0.008) in contrast to AFP levels that were higher among non-responders (P<0.001). Also, the SVR rate was 60% and 29.8% for those with serum AFP under and above the median value (5.5 ng/ml), respectively (OR  =  0.28, 95% CI  =  0.155–0.5; P<0.0001). Duration of treatment did not affect outcome where, SVR was achieved in 19/47 (40.4%) of patients treated for 24 weeks, versus 72/153 (47.1%) of those treated for 48 weeks with no statistically significant difference (p = 0.424). Regarding on treatment viral kinetics, rapid virological response (RVR) rate was (27%) defined as non-detectable RNA at the 4^th^ week of treatment and was significantly different among the two groups (P<0.0001). Among those, 40 out of 54 RVR patients achieved SVR (74% of RVR patients) and 51 out of 146 non RVR patients achieved SVR (34.9% of non RVR patients)

While baseline AST, ALT, leucocytes count, HOMA-IR, grade of fibrosis, viral load and other parameters were statistically non-significant between the two groups.

### Genotypes distribution for *IL28B* (rs12979680), *PD-1*.*3* (rs11568821) and *CTLA-4 49A/G* (rs231775) genes in patients

Table 2 describes the genotypic frequencies exhibited by the studied genes in the SVR versus NR group. Distributions complied with the Hardy–Weinberg equilibrium.

**Table 2:** Genotypes distribution for *IL28B (rs12979680)*, *PD-1*.*3 (rs11568821) and CTLA-4 49A/G* (rs231775) genes.

Genotype	Total n = 200	Responders	Non-responders	*p* value
		n = 91	n = 109	
***IL28B* C/C, n (%)**	70 (35%)	56 (61.5%)	14 (12.8%)	<0.001*
**C/T, n (%)**	98 (49%)	31 (34.1%)	67 (61.5%)	
**T/T, n (%)**	32 (16%)	4 (4.4%)	28 (25.7%)	
***PD-1*.*3* G/G, n (%)**	129 (64.5%)	51 (56.04%)	78 (71.56%)	0.028*
**G/A, n (%)**	65 (32.5%)	35 (38.46%)	30 (27.52%)	
**A/A, n (%)**	6 (3%)	5 (5.49%)	1 (0.92%)	
***CTLA-4* A/A, n (%)**	87 (43.5%)	32 (35.2%)	55 (50.46%)	0.019*
**A/G, n (%)**	88 (44%)	42 (46.1%)	46 (42.2%)	
**G/G, n (%)**	25 (12.5%)	17 (18.7%)	8 (7.34%)	

*Denotes p-values below the level of 0.05 which are considered significant

Regarding IL28B, 80% (56/70) of patients with C/C genotype achieved SVR, compared to only 26.9% (35/130) in patients with T* genotype.

As for *PD-1*.*3*, among the A* genotype carriers, 56.3% (40/71) were able to achieve SVR, while 39.5% (51/129) of the G/G genotype were responders.

Meanwhile, analysis of *CTLA-4* revealed significantly higher response rate of 68% (17/25) achieved in GG genotype alone and 52.2% (59/113) among G* genotypes in comparison to 36.7% among the A/A.

### Predictors of a sustained response

To evaluate the clinical applicability of individual SNPs, as well as other pretreatment parameters, the predictive ORs for each SNP between SVR and NR were calculated.

First, univariate logistic regression analysis showed no significant role of the patient’s age, BMI, sex, baseline viral load, ALT, or HOMA-IR on treatment outcome. On the other hand, pretreatment higher AFP and lower platelets count seemed to have a negative effect on treatment response and on treatment RVR was a strong predictor of SVR. With respect to *IL28B*, patients with C/C genotype had higher SVR than patients with non-C/C genotypes (OR 10.86; 95% CI 5.378–21.92; P <0.0001). Additionally, better SVR was obtained with A* genotypes (OR = 1.973; 95%CI: 1.097–3.55; P = 0.023) and G* genotypes (OR = 1.948; 95%CI: 1.1–3.3449; P = 0.022) of *PD-1*, and *CTLA-4* genes respectively as shown in Table 3.

**Table 3:** Predictors of SVR using univariate and multivariate logistic regression analysis.

	Univariate analysis		Multivariate analysis	
	OR (95%CI)	P	OR (95%CI)	P
**RVR**	5.32 (2.65–10.69)	<0.0001		
**AFP**	0.91 (0.86–0.96)	0.001	0.931(0.87–0.98)	0.016*
**PLT**	1.007 (1.002–1.012)	0.009	1.003(0.997–1.009)	0.355
***IL28B*CC (vs CT/TT)**	10.86 (5.37–21.92)	<0.0001	12.592 (5.65–28.03)	<0.001*
***PD-1* (GA+AA) vs GG**	1.97 (1.09–3.55)	0.023	1.49 (.71–3.11)	0.281
***CTAL-4* (GG+AG) vs AA**	1.94 (1.1–3.34)	0.022	2.07 (1.01–4.24)	0.045*

>1 odds ratio indicated that this factor was associated with an SVR to the treatment.

*Denotes p-values below the level of 0.05 which are considered significant

Furthermore, on separating the 20 patients (10%) who relapsed from NR, univariate analysis among the three groups revealed statistically significant differences between patients who reached SVR and who relapsed only for *IL28B* CC vs CT+TT (OR = 9; 95%CI: 2.475–33.21; P <0.002), but not between those who relapsed or NRs (OR = 1.25; 95%CI: 0.3146–4.977; P = 0.75), which suggests that the *IL28B* could not be used to accurately distinguish patients who relapse from those with NR.

Multivariate logistic regression analysis was done to identify independent pretreatment factors that contribute significantly to the prediction of the therapeutic outcome. Covariates with exception on-treatment variable RVR of p < 0.05 at univariate analysis were included in a multivariate model to determine independent determinants.

According to this analysis the AFP level, *IL28B* C/C genotype, and *CTLA-4* G* genotypes were significantly associated with SVR and were observed to be significant predictors of SVR. The most informative pretreatment markers to predict SVR of HCV treatment outcome in order of strength were, *IL28B* polymorphism, AFP and CTLA-4 polymorphism. Additionally, when *CTLA-4* was added to *IL28B CC group*, SVR rate increased in C/C carriers from 80% to 90% when G* genotype was present, and decreased to 66.6% when A/A was present (p = 0.03).

Accordingly, a regression model is built to estimate the probability (P) of SVR using the following formula:

$$p=\frac{e^{(}{}^{-0.853+\left(-0.086*AFP\right)+\left(2.457*IL28B\right)+\left(0.823*CTAL4\right))}}{1+e^{(}{}^{-0.853+\left(-0.086*AFP\right)+\left(2.457*IL28B\right)+\left(0.823*CTAL4\right))}}$$

Example for using the formula: for a patient with AFP = 1.2ng/ml, *CTAL*4: AG = favorable allele = 1, *IL28B*: CC = favorable allele = 1; P of SVR = 0.91 which is >0.436 so patient will probably achieve SVR.

**Fig 1:**
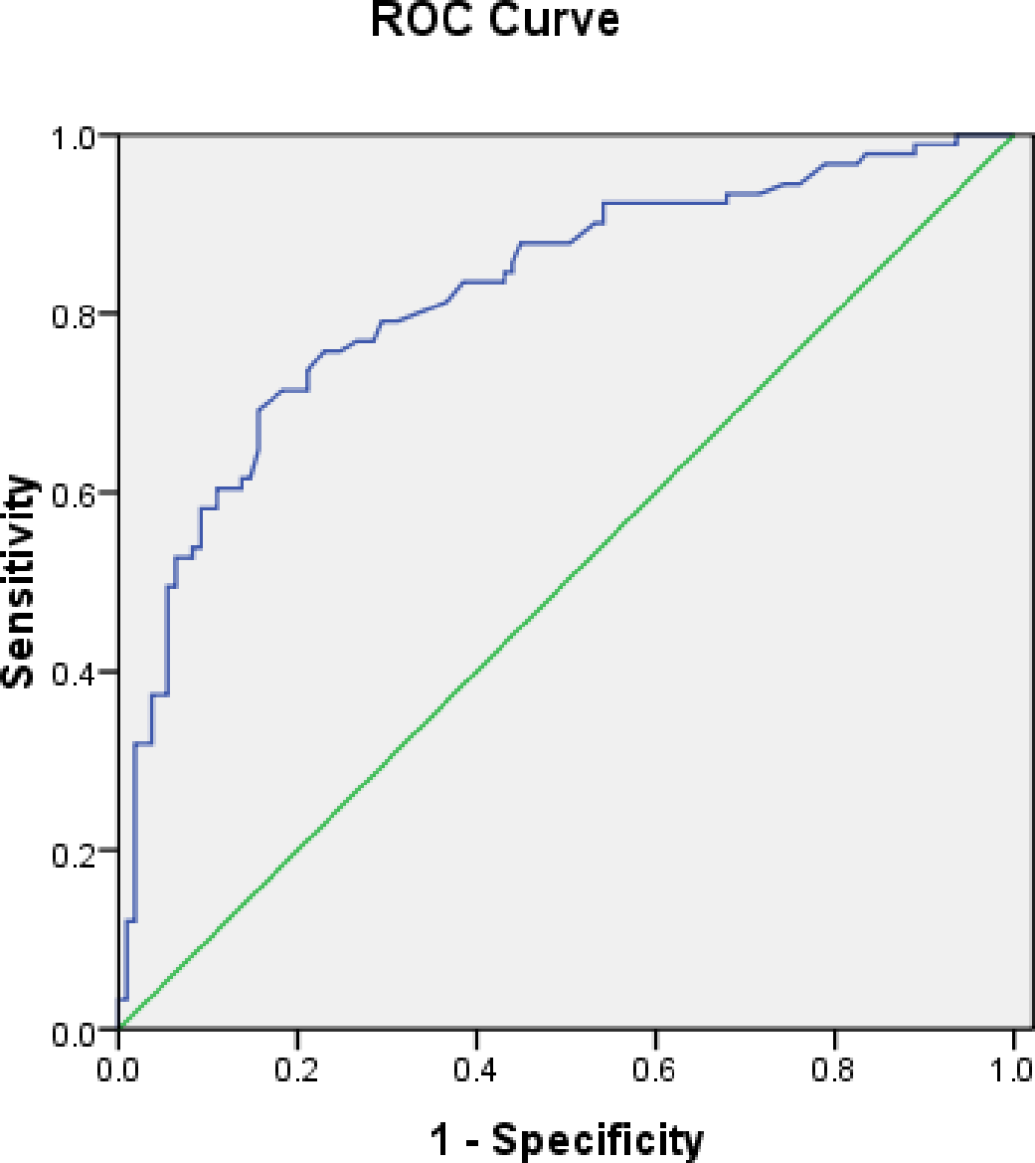
ROC curve provided by the model constructed to establish the predictive value for SVR. Area under the receiver-operating curve = 0.822 (95% CI = 0.76–0.88).

The ROC was plotted in accordance with the same model to establish specificity and sensitivity values (Fig 1). The best cut-off value for this model was 0.436 that provided a sensitivity of 71.4%, a specificity of 81.7%, PPV = 76.5% and NPV = 77.4% for predicting SVR.

## Discussion

Despite aggressive programs toward education, care, and treatment over the past years, Egypt still faces the largest burden of HCV infection in the world with a 10% prevalence of chronic infection among persons aged 15–59 years, predominantly genotype 4 [25]. The addition of DAAs to PEG-IFN + RBV, for HCV genotype 1 patients is under trial and evaluation for worldwide implementation [26]. New DAAs are now the new hope for chronic HCV patients. Six treatment options are available in 2015 for patients infected with HCV genotype 4, including 2 IFN-containing regimens and 4 IFN-free regimens. In settings where DAAs are not available, the combination of PegIFN-α and ribavirin remains acceptable [27]. Nevertheless, fear of resistance as most DAAs possess a low genetic barrier to resistance, poor response in prior null responders especially those with cirrhosis, non-eligibility for many patients [28] and affordability are major concerns [29]. Indeed, a number of Egyptians started new DAA regimen (with/out IFN/RBV), including (a) ledipasvir-sofosbuvir for 12 weeks, (b) ombitasvir-paritaprevir-ritonavir plus ribavirin for 12 weeks, or (c) sofosbuvir plus ribavirin for 24 weeks [27], with the government covering expenses of 80% of patients, treating 5 million patients of an estimated 11 million infected people will still cost Egypt $4·5 billion of the $5.4 billion total health budget for 2016–17 [30, 31]. A recent study indicated that Pegasys may be a superior choice of interferon therapy under low socioeconomic conditions [32]. Thus, in Egypt, PEG-IFN remains an integral part of treatment [33]. However, in our analysis, the SVR rate was 45.5%, which is similar to that reported by other studies ranging from 47% to 54% [34, 35].

Since a significant number of patients will fail to respond or will experience significant side effects, identification of host and viral factors predicting treatment outcome is of major interest [36]. However, studies about predictors of response within populations infected with HCV genotype 4 especially in Egypt are still scarce [37]. Various studies with albeit controversies had evaluated viral and host factors in relation to treatment response. In this study we analyzed some of the potential pretreatment factors that could be associated with the response to the therapeutic regimen. Preliminary analyses showed a non-significant difference in SVR between males and females and between patients with a low and high viral load nor fibrosis stage or presence of cirrhosis. We did not observe a significant difference in age, BMI, nor baseline ALT between SVR and NR groups. In contrast to Ogawa et al [15], who found HOMA-IR to be negatively associated with treatment response, our study did not find an association.

Studies pointed out to the role of thrombocytopenia in treatment failure and that attention should be paid to diagnosing potential thrombocytopenia in the treatment of chronic HCV patients [38]. Platelets count in our study was a predictor of treatment response; however this correlation did not persist in multivariate regression analysis. Significant elevations in AFP have been encountered in non-hepatic malignancies as chronic HCV in Egyptians [39]. Similarly, in our study, median AFP levels was 5.5 ng/ml ranging from 0.5 to 30 ng/ml, with median of 3 ng/ml in SVR group versus 7 ng/ml in NR, and with higher SVR rates achieved among patients with AFP below the median level. The only clinical pretreatment factor we found to be persistently negatively correlated to SVR was; AFP level, hence approving with recent studies of AFP in prediction of treatment response in Egyptian population [16, 37]. Interestingly recent studies pointed to correlation of liver fibrosis regression and low post treatment AFP levels after SVR [40].

Regarding host genetic factors, assessment of *IL28B* (rs12979680) polymorphism in our study revealed a 2–3 fold greater chance of SVR in patients carrying the C/C genotype than in patients with the T* genotype, which comes in accordance with numerous lines of evidence that have solidified the association between the IL28B C/C genotype and SVR in patients with chronic HCV [16, 41]. It is postulated that this SNP has effects on the binding of different transcription factors resulting in reduced expression of IL28B, and IFN- stimulated genes (ISGs) expression which may modulate the response to IFN. It was suggested that patients with CC genotype had low basal levels of hepatic ISG expression, and therefore when stimulated with IFN showed greater up regulation of ISGs and hence better treatment response [42].

Although the *IL28B* SNP is currently the best single pretreatment predictor of SVR as revealed by GWAS, not all patients with the favorable genotype achieve SVR, and some patients without it are nonetheless able to achieve SVR. Therefore, this SNP alone might not be sufficiently discriminative to advise a course of treatment [43] and useful prediction models taking into account other host genetic factors that might influence outcome of treatment are warranted [36].

In this setting, this is the first study that aimed to analyze the role of intronic *PD-1*.*3* (rs11568821) and *CTLA-4* (rs231775) genetic polymorphisms in response to IFN/RBV treatment of chronic HCV in Egyptian patients. The corresponding molecules encoded by these genes are normally expressed on the Treg surface producing a negative signal and thus preventing T cell activation [44]. It has been postulated that polymorphic sites at genes encoding these regulatory molecules can unbalance the immune regulation. They have been extensively studied in several immune relevant diseases including systemic lupus erythematosus [19], multiple sclerosis [45], rheumatoid arthritis [46], grave’s disease [47], type1 diabetes [48] and others. However, limited studies until now have been performed within chronic HCV infection.

Distribution of *PD-1* and *CTLA-4* in our study showed A/A and G/G to be mutant rare alleles respectively. Both *PD-1* and *CTLA-4* polymorphisms were found to be associated with treatment response in the univariate analysis, however in contrast to study by Vidal-Castineira, et al where *PD-1*.*3* increased the prediction of *IL28B* in genotype 1, *PD-1*.*3* was significant alone but when combined with other SNPs and factor was not among the strongest predictors [23]. While, *CTLA-4* was associated with treatment response in multivariate analysis, where the presence of favorable G* genotypes conferred greater rate of response. A recent study revealed spontaneous HCV clearance to be higher in patients carrying the *CTLA-4* G allele [49]. One of the postulated mechanisms for HCV chronicity is the high expression of PD-1 and CTLA-4 on HCV-specific T-cell [50]. In contrast to autoimmune diseases; in which the favorable alleles are those associated with higher PD-1 and CTLA-4 levels, polymorphisms associated with diminished CTLA-4 expression in HCV infections were found to correlate to improved viral clearance [51].

Our results showed that the most relevant pretreatment predictors to treatment response in order of strength are: *IL28B* polymorphism, AFP level, and *CTLA-4* polymorphism. Additionally *CTLA-4* G allele was found to significantly increase SVR rate from 80% to 90% in patients carrying *IL28* C/C and *CTLA-4* G* genotype, while decrease SVR to 66.6% when A/A genotype is present and could be further evaluated for consideration in treatment prediction. Arguably, the predictive power of genetic markers ranges vastly across different reports even within a highly homologous genetically population, accordingly replication and meta-analyses of such investigations across and within populations with different ethnic background is highly warranted and could help in establishment of prediction models [52]. The most obvious application of genotype testing in this study could be identifying subjects who might profit the most of the addition of a protease inhibitor to PEG-IFN/RBV. A potential approach could be to immediately add a DAA to PEG-IFNa/RBV in patients with a non-favorable predicted outcome, whereas treat those with favorable predicted outcome initially with PEG-IFN/RBV alone and DAAs could be made available as add-on treatment for those not achieving initial RVR or willing to pay. Such an approach would hopefully safely reduce overall costs. Further studies investigating the possible role of these polymorphisms in predicting response to DAA based triple therapy are required.

Hopefully, tailoring treatments to target potential responders, instead of generalized, universal treatment strategies, will be of economic benefit but, more importantly, will have substantial benefits for patients, leading to quick recovery and avoiding multiple ‘trial-and-error’ treatments [53].

## Conclusion

Since PEG-IFN/RBV is hampered by its long duration and the high burden of side effects, even more accurate predictors of outcome would help clinicians optimize treatment plans and duration. The aim of this study was to investigate for the first time the *PD-1*.*3 and CTLA-4* genetic polymorphism to predict SVR in HCV infected Egyptian and to emphasize the role of *IL28B* gene patients collectively with other potential pretreatment viral and host clinical parameters. In conclusion, for HCV genotype 4 patients, the *IL28B* CC (rs12979680) genotype, AFP level and *CTLA-4* (rs231775) G* genotypes are independent pretreatment predictors of SVR for patients treated with PegIFNα-2a and RBV.
